# Trait correlated expression combined with expression QTL analysis reveals biological pathways and candidate genes affecting water holding capacity of muscle

**DOI:** 10.1186/1471-2164-9-367

**Published:** 2008-07-31

**Authors:** Siriluck Ponsuksili, Elisabeth Jonas, Eduard Murani, Chirawath Phatsara, Tiranun Srikanchai, Christina Walz, Manfred Schwerin, Karl Schellander, Klaus Wimmers

**Affiliations:** 1 Research Institute for the Biology of Farm Animals (FBN), Research Group 'Functional Genomics', Wilhelm-Stahl-Allee 2, 18196 Dummerstorf, Germany; 2 Research Institute for the Biology of Farm Animals (FBN), Research Unit 'Molecular Biology', Wilhelm-Stahl-Allee 2, 18196 Dummerstorf, Germany; 3 Institute of Animal Science, Animal Breeding and Husbandry Group, University of Bonn, Endenicher Allee 15, 53115 Bonn, Germany

## Abstract

**Background:**

Leakage of water and ions and soluble proteins from muscle cells occurs during prolonged exercise due to ischemia causing muscle damage. Also *post mortem *anoxia during conversion of muscle to meat is marked by loss of water and soluble components from the muscle cell. There is considerable variation in the water holding capacity of meat affecting economy of meat production. Water holding capacity depends on numerous genetic and environmental factors relevant to structural and biochemical muscle fibre properties a well as *ante *and *post *slaughter metabolic processes.

**Results:**

Expression microarray analysis of M. *longissimus dorsi *RNAs of 74 F2 animals of a resource population showed 1,279 transcripts with trait correlated expression to water holding capacity. Negatively correlated transcripts were enriched in functional categories and pathways like extracellular matrix receptor interaction and calcium signalling. Transcripts with positive correlation dominantly represented biochemical processes including oxidative phosphorylation, mitochondrial pathways, as well as transporter activity. A linkage analysis of abundance of trait correlated transcripts revealed 897 expression QTL (eQTL) with 104 eQTL coinciding with QTL regions for water holding capacity; 96 transcripts had *trans *acting and 8 had *cis *acting regulation.

**Conclusion:**

The complex relationships between biological processes taking place in live skeletal muscle and meat quality are driven on the one hand by the energy reserves and their utilisation in the muscle and on the other hand by the muscle structure itself and calcium signalling. Holistic expression profiling was integrated with QTL analysis for the trait of interest and for gene expression levels for creation of a priority list of genes out of the orchestra of genes of biological networks relevant to the liability to develop elevated drip loss.

## Background

Prolonged exercises may lead to damage of muscle fibres with leakage of water, ions and proteins, in particular muscle specific enzymes, whose serum levels are diagnostic for muscle injury [1]. However the mechanisms that underlie the development of cellular muscle damage after exercise are not yet clarified. The extend of physical burden as well as nutritional and metabolic aspects, genetics and temperature affect the degree of damage, that is due to an imbalance of energy and oxygen demands and supply of the muscle cells [2-5]. Similarly, at *post mortem *termination of nutrient and energy supply and anoxia occurs. *Post mortem *anaerobe energy production by glycolysis stops at low pH and finally energy supply collapses marked by increased cytoplasmatic Ca^2+ ^levels and activation of Ca^2+ ^dependent intracellular processes. The conversion of muscle to meat is thus marked by the activity of proteins of the anaerobic energy and calcium metabolism, lactacidosis, assembly of actin-myosin-complexes and proteolysis. This is accompanied by leakage of the muscle cells and loss of water, ion and proteins. There is considerable variation in the amount of fluid released from the muscle during maturation to meat that might reflect difference in the sensitivity to metabolic imbalance and physical stressors of various genotypes. These biochemical processes play an important role not only in muscle injury but also in meat quality in pork industry. Water holding capacity (WHC) of pork is an important aspect of palatability that affects overall quality and acceptability of meat and is a consistent problem in the pork industry for many years [6,7]. Water-holding capacity can be measured in form of drip loss. Drip development during storage of meat is principally caused by shrinkage of myofibrils due to changes of energy reserves, pH and temperature *post mortem *[8]. Heritability estimates for drip loss vary from 0.08–0.30 depending on the method of drip measurement or breed [9-13]. Biological mechanisms and the genetic background underlying variation in drip are not fully understood.

A genome scan is the most general approach to identify genomic regions exhibiting quantitative trait loci (QTL), classically for complex phenotypic characteristics that vary in degree and can be attributed to effects of many gene (subsequently termed pheneQTL = pQTL). QTL for WHC were mapped in many regions of porcine chromosomes [14-17]. QTL regions are generally large and contain several putative causal genes. Combining microarray data with quantitative trait loci (pQTL) linkage studies offers new options of understanding the biology at a global level and the genetic factors affecting the trait of interest. Integration of positional and functional information facilitates focussing on most relevant candidate genes in each pQTL region [18]. QTL analysis of expression levels of a gene identifies genomic regions, which are likely to contain at least one causal gene with regulatory effect on the expression level, termed expression QTL, eQTL [19,20]. In order to identify genes and pathways with multiple evidence of their role in the biology of a trait, it is proposed here to combine (1) information on pQTL with analysis of (2) trait correlated expression and with (3) mapping of eQTL for the corresponding trait dependent regulated genes. Under the assumption that genes with trait correlated expression levels belong to pathways or networks relevant for the control of the trait, correlation analysis of microarray expression data and records of WHC, measured as drip loss, reveals a list of functional candidate genes. Functional annotation allows identification of biological pathways and offers an insight into the biological processes causing variation in the genetically based trait, WHC. Information of the expression study merged with results of a pQTL study for the trait drip loss performed in the same population already down scales the list of primary candidate genes. Further, adding eQTL analyses for transcripts showing trait dependent expression enables addressing genes, which show trait associated expression, map to pQTL regions, and exhibit *cis *regulation. These genes are functional positional candidate genes likely to exhibit polymorphisms affecting their own expression and by this the phenotypic trait drip loss, i.e. they are likely to be causal genes in the pQTL of that trait.

## Results

**Figure 1:**
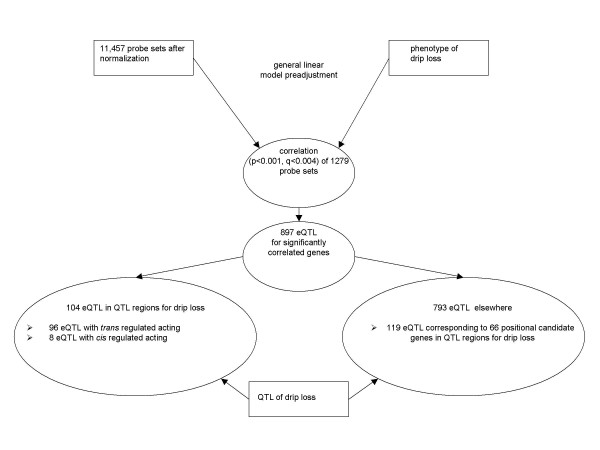
**Strategy of identification of candidate genes for WHC**. Genes rated present after normalization were included in the statistical analysis. Both expression and phenotype data were adjusted using the general linear model before performing Pearson correlation analysis. Genes with trait correlated expression were included in the eQTL analysis. Genes with significant eQTL were assigned *cis *regulated if the genes' position matches the position of its eQTL, others were considered *trans *regulated. Also the position of the genes relative to QTL for drip was taken into account.

Expression profiling and eQTL analysis were conducted on 74 F2 animals of a resource population with previously identified pQTL for drip [21]. The 74 animals were chosen to give a good representation of the population in terms of families and genotypes at the major pQTL [22]. Using Affymetrix Porcine Genome Arrays, 23,256 expression measurements were obtained from each M. *longissimus dorsi *RNA samples of these animals representing 11,265 unique genes according to the annotation reported by Tsai et al. [23]. After processing the Affymetrix CEL files with MAS5, where a 'present call' is assigned, the pre-selected data set was further analyzed with the more sophisticated hybrid algorithm PLIER [24-27]. This revealed 11,453 probe sets for further. The overall strategy to identify functional positional candidate genes is shown on Figure 1.

### Correlation of transcript abundance and drip loss

**Figure 2:**
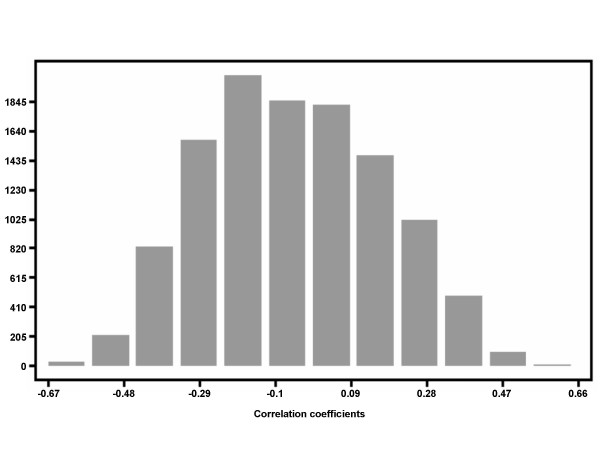
Histogram of the distribution of pair wise correlation coefficients of expression value and drip loss.

The normalized expression data and drip loss phenotypes were pre-adjusted for systematic effects of family and treatment/environment using a general linear model. Pearson correlation was calculated between each of the 11,453 gene expression values and drip loss phenotypes. A histogram of pair wise correlation coefficients of expression value and drip loss is shown on Figure 2. A total of 1,279 genes were significantly associated at *p *≤ 0.001 corresponding to *q *≤ 0.004, with 601 genes showing negative correlation and 678 genes showing positive correlation of their transcript abundance with drip loss. The lists of coefficients of correlation (r) between drip loss and expression levels, p-values, as well as corresponding q-values are shown in supplementary table 1 [see Additional file 1]. The correlations ranged between l0.37-0.67l.

### Biological pathway associated with drip loss

We tested the list of significantly positive and negative correlated genes for enrichment in functional annotation groups as defined in the Gene Ontology (GO) and Kyoto Encyclopedia of Genes and Genomes (KEGG) databases [28]. Out of 1279 probes sets 1076 had records in the GO database. GO categories of genes with expression levels negatively correlated with WHC, i.e. positively correlated to drip loss, are shown in Table 1. Five functional groups were found significant (median of EASE Score ≤ 0.05) including genes related to receptor and signal transducer activity, non-membrane-bound organelles, cytoskeleton, plasma membrane, and cell communication and signalling. Two KEGG pathways were also significant (EASE Score ≤ 0.05) 'extracellular matrix receptor interaction' and 'calcium signalling pathway'. The lists of genes of both pathways as well as the correlation coefficient with drip loss are shown in Table 2 and 3. Among the genes with negative correlation of expression level with drip loss, functional groups represented were mitochondrial genes, electron and ion transporter activity, and protein metabolism (Table 4). The KEGG pathway 'oxidative phosphorylation', which belongs to the functional group of transporters activity, was also significant (EASE Score = 1.26E-06). Twenty genes in this pathway were found significantly correlated with drip loss (Table 5).

**Table 1:** GO categories of genes with positively correlated expression with drip loss

Functional Group 1	Median: 0.005	Number of genes	% of genes in pathways	EASE Score
GOTERM_MF_ALL	receptor activity	55	8.91	0.000
GOTERM_MF_ALL	signal transducer activity	84	13.61	0.006
GOTERM_MF_ALL	transmembrane receptor activity	28	4.54	0.009
				
Functional Group 2	Median: 0.006			
GOTERM_CC_ALL	intracellular non-membrane-bound organelle	76	12.32	0.006
GOTERM_CC_ALL	non-membrane-bound organelle	76	12.32	0.006
				
Functional Group 3	Median: 0.011			
GOTERM_BP_ALL	cytoskeleton organization and biogenesis	28	4.54	0.003
GOTERM_MF_ALL	actin binding	22	3.57	0.003
GOTERM_MF_ALL	cytoskeletal protein binding	28	4.54	0.005
GOTERM_BP_ALL	actin filament-based process	16	2.59	0.011
GOTERM_CC_ALL	cytoskeleton	44	7.13	0.013
GOTERM_BP_ALL	actin cytoskeleton organization and biogenesis	15	2.43	0.014
GOTERM_CC_ALL	actin cytoskeleton	17	2.76	0.061
				
Functional Group 4	Median: 0.018			
GOTERM_CC_ALL	plasma membrane	65	10.53	0.006
GOTERM_CC_ALL	integral to plasma membrane	43	6.97	0.018
GOTERM_CC_ALL	intrinsic to plasma membrane	43	6.97	0.020
				
Functional Group 5	Median: 0.011			
GOTERM_BP_ALL	cell communication	114	18.48	0.011
GOTERM_BP_ALL	signal transduction	107	17.34	0.011
GOTERM_BP_ALL	intracellular signaling cascade	52	8.43	0.046

**Table 2:** Genes of extracellular matrix receptor pathway positively correlated with drip loss

AFFY_ID	r	p-value	q-value	gene name (gene symbol)
Ssc_24909_1_S1_at	0.413	0.0003	0.0017	laminin, alpha 4 (LAMA4)
Ssc_8843_1_A1_at	0.397	0.0005	0.0023	fibronectin 1 (FN1)
Ssc_3902_1_S1_at	0.476	<.0001	0.0001	septin 5 (PNUTL1)
Ssc_4345_1_S2_at	0.386	0.0007	0.0028	collagen, type IV, alpha 1 (COL4A1)
Ssc_16589_1_S1_at	0.380	0.0008	0.0031	collagen, type VI, alpha 3 (COL6A3)
Ssc_1099_1_S1_at	0.398	0.0004	0.0020	laminin, gamma 1 (LAMC1)
Ssc_1091_3_A1_at	0.472	<.0001	0.0001	collagen, type I, alpha 1 (COL1A1)

**Table 3:** Genes of calcium signalling pathway positively correlated with drip loss

AFFY_ID	r	p-value	q-value	gene name (gene symbol)
Ssc_22248_1_A1_at	0.478	<.0001	0.0001	guanine nucleotide binding protein, alpha stimulating complex locus (GNAS)
Ssc_17453_1_S1_at	0.389	0.0006	0.0025	ATPase, Ca++ transporting, plasma membrane 4 (ATP2B4)
Ssc_4203_1_S1_at	0.379	0.0009	0.0033	v-erb-b2 erythroblastic leukemia viral oncogene homolog 3 (ERBB3)
Ssc_7883_1_A1_at	0.377	0.0009	0.0033	oxytocin receptor (OXTR)
Ssc_25651_1_S1_at	0.446	<.0001	0.0001	protein phosphatase 3 (formerly 2B), catalytic subunit, beta isoform (PPP3CB)
Ssc_22641_1_S1_at	0.489	<.0001	0.0001	ATPase, Ca++ transporting, cardiac muscle, slow twitch 2 (ATP2A2)
Ssc_55_1_S1_at	0.664	<.0001	0.0001	epidermal growth factor receptor (EGFR)
Ssc_8_1_S1_at	0.395	0.0005	0.0023	ryanodine receptor 1 (RYR1)

**Table 4:** GO categories of genes with negatively correlated expression with drip loss

Functional Group 1	Median: 1.69E-4	Number of genes	% of genes in pathways	EASE Score
GOTERM_CC_ALL	mitochondrion	63	11.89	8.28E-09
				
Functional Group 2	Median: 2.35E-4			
GOTERM_MF_ALL	electron carrier activity	17	3.21	9.21E-07
GOTERM_MF_ALL	NADH dehydrogenase activity	13	2.45	2.26E-06
GOTERM_MF_ALL	carrier activity	35	6.60	3.57E-06
GOTERM_MF_ALL	sodium ion transporter activity	13	2.45	4.24E-06
GOTERM_MF_ALL	oxidoreductase activity	15	2.83	5.05E-06
GOTERM_MF_ALL	hydrogen ion transporter activity	20	3.77	4.90E-05
GOTERM_MF_ALL	inorganic cation transporter activity	20	3.77	8.29E-05
GOTERM_MF_ALL	primary active transporter activity	22	4.15	9.72E-05
GOTERM_MF_ALL	electron transporter activity	25	4.72	1.29E-04
GOTERM_MF_ALL	metal ion transporter activity	14	2.64	3.41E-04
GOTERM_MF_ALL	cation transporter activity	30	5.66	6.26E-04
GOTERM_MF_ALL	ion transporter activity	33	6.23	0.002
GOTERM_MF_ALL	transporter activity	60	11.32	0.007
GOTERM_BP_ALL	generation of precursor metabolites and energy	32	6.04	0.032
				
Functional Group 3	Median: 0.050			
GOTERM_BP_ALL	protein metabolism	131	24.72	0.002
GOTERM_BP_ALL	cellular protein metabolism	121	22.83	0.003
GOTERM_BP_ALL	cellular metabolism	244	46.04	0.005
GOTERM_BP_ALL	cellular macromolecule metabolism	121	22.83	0.005
GOTERM_BP_ALL	macromolecule metabolism	168	31.70	0.007
GOTERM_BP_ALL	metabolism	255	48.11	0.013
GOTERM_BP_ALL	cellular process	338	63.77	0.051

**Table 5:** Genes of with oxidative phosphorylation pathway negatively correlated with drip loss

AFFY_ID	r	p-value	q-value	gene name (symbol)
Ssc_886_1_S1_at	-0.381	0.0008	0.0035	cytochrome c-1 (CYC1)
Ssc_2028_1_S1_at	-0.400	0.0004	0.0022	ATPase, H+ transporting, lysosomal 14 kDa, V1 subunit F (ATP6V1F)
Ssc_26100_1_S1_at	-0.438	<.0001	0.0001	NADH dehydrogenase (ubiquinone) 1 beta subcomplex, 7 (NDUFB7)
Ssc_922_2_S1_at	-0.449	<.0001	0.0001	NADH dehydrogenase (ubiquinone) flavoprotein 2 (NDUFV2)
Ssc_3869_1_A1_at	-0.451	<.0001	0.0001	NADH dehydrogenase (ubiquinone) 1, subcomplex unknown, 1 (NDUFC1)
Ssc_15103_1_S1_at	-0.543	<.0001	0.0001	NADH dehydrogenase (ubiquinone) Fe-S protein 6 (NDUFS6)
Ssc_22694_1_S1_at	-0.413	0.0003	0.0019	NADH dehydrogenase (ubiquinone) 1 beta subcomplex, 6 (NDUFB6)
Ssc_21308_2_S1_at	-0.387	0.0007	0.0033	cytochrome c oxidase assembly protein (COX10)
Ssc_2184_1_S1_at	-0.415	0.0002	0.0015	cytochrome c oxidase subunit VIa polypeptide 2 (COX6A2)
Ssc_2957_1_S1_at	-0.500	<.0001	0.0001	ATP synthase, H+ transporting, mitochondrial F0 complex, subunit G (ATP5L)
Ssc_1219_1_S1_at	-0.439	<.0001	0.0001	ATP synthase, H+ transporting, mitochondrial F0 complex, subunit d (ATP5H)
Ssc_17183_1_S1_at	-0.445	<.0001	0.0001	ATP synthase, H+ transporting, mitochondrial F1 complex, delta subunit (ATP5D)
Ssc_1108_1_S1_at	-0.398	0.0004	0.0022	NADH dehydrogenase (ubiquinone) 1 alpha subcomplex, 8 (NDUFA8)
Ssc_6891_1_S1_at	-0.392	0.0006	0.0029	NADH dehydrogenase (ubiquinone) 1 beta subcomplex, 4 (NDUFB4)
Ssc_23542_1_A1_at	-0.406	0.0003	0.0019	NADH dehydrogenase (ubiquinone) 1 beta subcomplex, 11 (NDUFB11)
Ssc_20956_1_S1_at	-0.401	0.0004	0.0022	NADH dehydrogenase (ubiquinone) 1 alpha subcomplex, 3 (NDUFA3)
Ssc_1287_1_S1_at	-0.382	0.0008	0.0035	NADH dehydrogenase (ubiquinone) 1 beta subcomplex, 9 (NDUFB9)
Ssc_3708_1_S1_at	-0.430	0.0001	0.0009	NADH dehydrogenase (ubiquinone) 1 alpha subcomplex (NDUFA12)
Ssc_1687_1_S1_at	-0.433	0.0001	0.0009	NADH dehydrogenase (ubiquinone) 1 beta subcomplex, 10 (NDUFB10)
Ssc_24943_1_S1_at	-0.466	<.0001	0.0001	NADH dehydrogenase (ubiquinone) 1 alpha subcomplex, 11(NDUFA11)

### Coincidence of eQTL and pQTL for drip loss

In order to scale down the list of candidate genes that can be derived from the previously done pQTL study and from the global analysis of trait correlated expression presented here we aimed to combine these approaches with an analysis of eQTL.

Classically, QTL analysis is applied for the identification of genes responsible for complex traits such as meat quality or growth traits (pQTL). Similarly, when the expression levels of genes are defined as a quantitative trait, QTL analysis can map the genetic determinants responsible for their transcriptional levels (eQTL). As described by Liu [21], four pQTL for drip loss were identified with line cross models on SSC2, SSC3, SSC5 and SSC18 between *SW2623-S0141*, *SW72-S0164*, *SW491-SW1482 *and *S0062-SWR414*, respectively. For half sib models, pQTL for drip were detected on SSC6 and SSC18 at position *S0035-S0087 *and *SWR414-SY31*. Combined line cross and half sib models revealed an additional drip loss pQTL on SSC4 in position *S0214-S0097 *(Liu et al., accepted).

**Figure 3:**
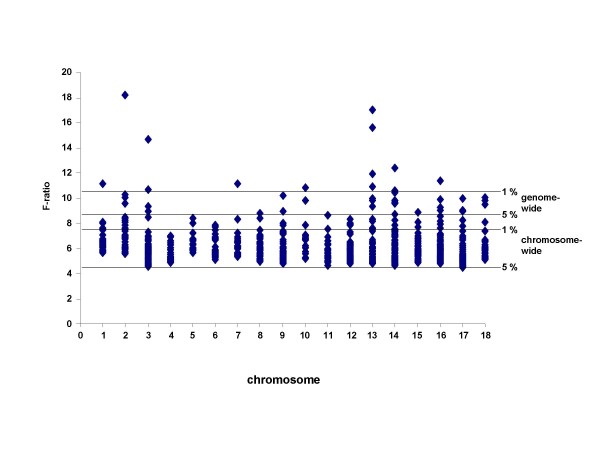
***F*-statistic of a total of 897 eQTL on all 18 porcine chromosomes**. The horizontal lines represent the respective significance thresholds.

A total of 1279 genes with significant correlation of transcript abundance to drip loss were selected for eQTL linkage mapping. Significance thresholds were determined by 10,000 permutations according to Churchill and Doerge [29] revealing 5% and 1% chromosome-wide significance levels as well as 5% and 1% genome-wide significance levels after Bonferroni correction for 18 autosomes of the haploid porcine genome. The 5% chromosome-wide threshold corresponds approximately to the suggestive linkage threshold proposed by Lander and Kruglyak [30]. In total the analysis revealed 897 eQTL with chromosome-wide significance at the p ≤ 0.05 level including 156 eQTL significant at the p ≤ 0.01 chromosome-wide level and 48 and 12 eQTL significant at genome-wide p ≤ 0.05 and p ≤ 0.01 levels, respectively [see Additional file 2]. The eQTL distribution on all chromosomes is shown in Figure 3. The F-value of eQTL ranged from 4.4 to 18.2 corresponding to LOD scores of 1.8 to 6.4 for different chromosomes. In total 104 significant eQTL were detected in the pQTL target regions for drip loss on SSC2, 3, 4, 5, 6, and 18 [see Additional file 3]. Additional 66 candidate genes mapping within the pQTL regions for drip loss on SSC 2, 3, 4, 5, 6 and 18 showed 119 eQTL in other genomic regions, thus indicating *trans *mode of regulation [see Additional file 4].

Mapping of eQTL to the gene itself indicates that *cis *changes are responsible for the different expression levels, whereas mapping positions of eQTL different from the position of the corresponding genes indicate *trans *regulation. By these definitions of *cis *and *trans *acting regulation out of 104 eQTL that coincided with pQTL for drip, 96 belong to genes that had *trans *acting regulation of transcription, 8 genes had *cis *acting transcriptional regulation (Table 6). For 7 out of these 8 *cis *regulated genes additional *trans *acting regulatory regions were found.

**Table 6:** Eight candidate genes with *cis *eQTL in the region of drip loss QTL of SSC2, 3, 4 and 6 and trait correlated expression

	eQTL			trait correlated expression			
			
Probe_Set_ID	SSC	POS [cM]	F	r	p-value	q-value	gene name (symbol)
Ssc.16645.1.S1_at	2	20	18.2	0.53	<.0001	9.44E-05	AHNAK nucleoprotein (AHNAK)
Ssc.2330.1.S1_at	2	35	6.5	-0.43	<.0001	8.77E-04	solute carrier family 3 (SLC3A2)
Ssc_3574_1_A1_at	3	0	9.4	0.56	<.0001	9.44E-05	mitogen-activated protein kinase kinase kinase kinase 4 (MAP4K4)
Ssc_10360_1_S1_at	3	0	5.1	0.51	<.0001	9.44E-05	hypothetical protein (LOC162073)
Ssc.20772.1.S1_at	3	37	5.2	-0.49	<.0001	1.03E-04	ubiquitin specific peptidase 39 (USP39)
Ssc_12110_1_S1_at	4	66	7.0	-0.58	<.0001	1.03E-04	papillary renal cell carcinoma (PRCC)
Ssc_5334_1_S1_at	6	42	7.5	-0.47	<.0001	1.03E-04	coenzyme Q9 homolog (CoQ9)
Ssc_4843_1_A1_at	6	46	5.1	0.45	<.0001	9.44E-05	Bardet-Biedl syndrome 2 (BBS2)

According to mapping information of Affymetrix probe sets accessible via the PigQTLdb about 300 porcine Affymetrix elements are located at the pQTL for drip loss on SSC2 between markers *SW2623-S0141 *[[31], ]. In this region 14 eQTL were mapped. Out of these 2 corresponded to genes that were under *cis *acting transcriptional control, Ssc.16645.1.S1 (*AHNAK*) and Ssc.2330.1.S1 (*SLC3A2*), while the other corresponded to genes that mapped elsewhere, thus indicating *trans *acting control. Because of the interesting functional links of *AHNAK *to WHC its position was confirmed by RH mapping. *AHNAK *was assigned to the SSC2 close to marker *SWR783 *(LOD score 17.8) in the interval of *SW2623 *and *S0141*. Quantification of transcripts of AHNAK and SLC3A2 by real time reverse transcription PCR (RT-PCR) revealed significant correlation with expression data obtained from microarray analysis (r = 0.6; p < 0.0001 and r = 0.4; p = 0.002, respectively). Moreover, trait correlated expression of AHNAK and SLC3A2 was confirmed (r = 0.3; p = 0.02 and r = -0.4; p = 0.004, respectively). Mapping of eQTL for AHNAK and SLC3A2 based on real time RT-PCR data fit results based on microarray data (marker interval *SW2623 *and *S0141; *F = 7 and F = 5, respectively; chromosome-wide significant at p ≤ 0.5). On SSC3, 545 probes sets were found between markers *SW72 *and *S0164*. 38 eQTL were detected in the drip pQTL region with three having *cis *acting transcriptional regulation: Ssc_3574_1_A1_at (*MAP4K4*), Ssc.20772.1.S1_at (*USP39*) and Ssc_10360_1_S1_at (hypothetical protein (*LOC162073*)). 14 eQTL were detected in the SSC4 pQTL region for drip and only one *cis *acting eQTL (Ssc_12110_1_S1_at (*PRCC)*) was found. No eQTL were detected in the SSC5 drip pQTL between markers *SW491 *and *SW1482*. 305 probes sets were found in the drip QTL region of SSC6. Out of 24 eQTL in that region, two eQTL indicated *cis *mode of regulation (Ssc_4843_1_A1_at (*BBS2*), Ssc_5334_1_S1_at (*COQ9*)). 110 probes sets were detected in the pQTL region of SSC18. All of 11 eQTL, which were detected in this region, showed *trans *acting regulation of expression.

## Discussion

WHC is influenced by many factors including genetic and environmental effects. We addressed the problem to dissect the genetic background of this complex trait by using the strategy of combining (1) the correlation of expression of many thousands of genes measured simultaneously by microarray technology with quantitative phenotypic data of drip loss, (2) mapping of QTL for the trait drip loss, and (3) mapping of QTL for the expression levels of genes with trait associated expression (Figure 1). QTL analyses provide information suitable to address positional candidate genes whereas trait associated expression studies reveal functional candidate genes. Taking both together, i.e. taking into account the localisation of functional candidate genes in QTL regions, enables to define functional positional candidate genes. Additional insight from eQTL analysis derives from three cases. (1) eQTL are detected within the pQTL but the functional candidate genes itself are located elsewhere, i.e. they are under *trans *control. These are genes that are likely to be trait dependent expressed due to hierarchically superior genes located in the pQTL that actually represent candidate genes (positional candidate genes). Here the eQTL analysis provides a link between functional and positional candidate genes. In this study 90 functional candidate genes were found with their corresponding 96 eQTL being situated within the previously detected pQTL. These genes point to biological pathways, which are relevant to the trait, and perhaps to causal genes underlying the QTL. However these genes themselves may either be not polymorphic, or the power of the pQTL analysis was not sufficient to detect them, or trait associated expression of these genes is rather an effect than a cause of variation. (2) For functional positional candidate genes being under *trans *control it can be speculated that the nature of variation affecting the phenotype is differential expression due to polymorphisms in hierarchically superior genes and different responsiveness of the candidate genes to regulatory mechanisms. Here the eQTL combined with pQTL and trait associated expression directs to biological pathways and genes relevant for the trait of interest. In total 66 positional functional candidate genes which corresponded to 119 eQTL with *trans *mode of expression regulation were found. (3) For genes categorized positional functional candidate genes, mapping of their corresponding eQTL in *cis *highlights them as genes showing variation with impact on the trait of interest and the expression level, indicating that the nature of the variation is likely a polymorphism in regulatory regions of the gene. Eight genes of this category were identified in this study. These genes are regarded as primary targets for further analysis.

However, it is important to mention that phenotypic variation may be due to genetic variation causing differential expression and/or structural variation of the gene products. The later are not addressed by eQTL analyses. Further, there are functional candidate genes that are under *cis *mode of transcriptional regulation, where there is no link between eQTL and pQTL analyses.

### Trait dependent expression analysis

The association between a quantitative phenotype and gene expression can be examined pair wise using a Pearson correlation coefficient between the expression of a single gene and a continuous phenotype. The approach of trait correlated expression analysis already proofs to be useful by many studies [32-34]. Kraft [33] used the within-family correlation analysis to remove the effect of family stratification. Here we used general linear models to account for systematic effects of family and environment on both drip phenotypes and expression levels in the correlation analysis. The pre-adjustment of individual phenotypes and expression levels increased the power to identify genetic effects compared to analyses conducted with raw data and revealed biologically meaningful relationship among the traits. Blalock et al. [34], considered correlation significant at *p *≤ 0.05 corresponding to false discovery rates of 20%. In this study, genes were considered for further analysis showing correlation coefficients between gene expression and drip loss of r ≥ 0.37, with *p *≤ 0.001 and corresponding *q *≤ 0.004.

### Biological categories and/or pathways of positively correlated genes

Currently, we do not completely understand the specific biochemical and/or biophysical mechanisms underlying differences in meat water holding capacity. The processes of muscle conversion to meat occurred in *post mortem *stage. One possible explanation for some of the variation that exists resides in the structure of the muscle cell itself. Most studies concentrated on *post mortem *process of drip [35,36]. In this study, transcript levels of muscle at slaughter were correlated with drip loss at *post mortem *meat stage in order to reveal insight into the biological processes that are initiated during life and thus are under genetic control and finally determine the liability to develop elevated drip loss. The mechanism underlying this liability trait may also be valid for the (patho-) physiological processes that take place during muscle damage due to biochemical and physical burden at prolonged exercises. Functional annotation analysis is essentially based on the extrapolation of pathway information and gene ontology data of human to the pig. Thus general cellular physiological processes are taken into account, whereas any pig-specific functional annotation data and in particular information on the physiology of the trait drip loss are not addressed during the automated bioinformatics analyses. However, the relevant knowledge has been taken into account in the biological interpretation of the results. The study revealed changes in genomic regulation of multiple cellular pathways that correlate with drip loss. The genes with positive correlation of transcript abundance and drip loss were genes of the group of receptor activity, non-membrane-bound organelle, cytoskeleton, plasma membrane and cell signal. Recently, many studies have shown that degradation of cytoskeleton and other structural proteins plays an important role in drip loss at *post mortem *[35,37-39]. As shown in this study the transcript abundance of genes of the cytoskeleton and other structural proteins increased with increasing drip loss. Extra cellular matrix proteins binding integrins and interacting with the cell cytoskeleton are important in controlling many steps in cell membrane-cytoskeleton attachments and in signalling pathways [40]. The degradation of integrin has been suggested to increase the drip channel formation between the cell and cell membrane and thus to be associated with drip loss during *post mortem *storage on pork [37,41]. The degradation of integrin may be due to the activity of the calpain system which requires high concentration of calcium for activation [41]. In this study, the enrichment of transcripts of extra cellular matrix receptor pathways among the positively drip correlated genes suggested that WHC may be involved with a breakdown of this extracellular matrix that activate the proteolytic system and thereby promote enzymatic degradation [42]. Calcium signalling pathways are very peculiar in nature. When there is an extracellular change, cells get the message either by introduction of calcium ions into cytoplasm or by evacuation to outside through ion channels. Increase in intranuclear Ca^2+ ^initiates gene expression and cell cycle procession, but also can activate degradative processes in programmed cell death or apoptosis [43]. Gene sets associated with calcium signalling pathways were enriched with decreasing water holding capacity. For example, epidermal growth factor receptor (EGFR) showed highest positive correlation with drip loss (r = 0.67, p < 0.0001). An early signal generated by the activation of EGFR upon ligand binding is a transient increase in the cytosolic concentration of free calcium ion ([Ca^2+^]_cyt_) [44]. Entry of extracellular Ca^2+^, and Ca^2+ ^release from intracellular stores, both appear to contribute to the generation of the EGF-mediated [Ca^2+^]_cyt _spike [45-47]. Early *post mortem *higher Ca^2+ ^concentration causes rapid contraction, an increase in the rate of muscle metabolism, and accelerated pH decline with resulting higher drip [38]. Another hypothesis is that higher Ca^2+ ^concentration present in muscle fibres early *post mortem *is a source for the activation of Ca^2+ ^dependent protease, phosphatases and phospholipases like the calpain system which influences drip production. Increased cytoplasmic Ca^2+ ^levels are also observed due to excessive exercises. This may initiate vicious cycles of cell degradation because of the Ca^2+ ^dependent activation of proteolytic enzymes such as calpain that by themselves digest structural elements of the muscle fibres leading to membrane damage, leakage of intracellular water and proteins and further accumulation of Ca^2+ ^[48]. Together, increase in transcript levels of genes involved in cytoskeleton, and extracellular matrix receptor pathways as well as calcium signalling pathways in muscle play an important role in final meat quality.

### Biological categories and/or pathways of negatively correlated gene

Though the energy metabolism is crucial for muscles, the biochemical processes involved in the change from aerobic metabolism *ante mortem *to anaerobic metabolism *post mortem*, which associates to drip loss, is not much investigated. The negatively correlated transcripts were enriched in mitochondrion, transporter activity and protein metabolism GO categories as well as oxidative phosphorylation pathway. A dominant role of mitochondria is the production of ATP by several different biochemical routes, i.e. via aerobe glycolysis and via oxidative phosphorylation. At the pre-slaughter stage in living animals with the presence of oxygen, aerobic processes take place. When oxygen is limited (*post mortem*) the glycolytic products will be metabolised by anaerobic respiration, a process that is independent of the mitochondria. A shift from aerobic to anaerobic metabolism – favouring the production of lactic acid – results in a pH decline *post mortem *and thereby influence the water holding capacity in muscle [49]. In our study, 63 transcripts belong to mitochondrion GO category and 20 transcripts belong to the oxidative phosphorylation pathway. The negative correlations with drip loss may indicate reduced activity of biochemical processes of ATP production via oxidative pathways in mitochondria of animals with high drip loss, reduced number of mitochondria in their muscle, i.e. higher content of glycolytic fibers, or reduced ATP reserves in the muscle.

Together, analysis of trait correlated expression revealed that the complex relationships between biological processes taking place in live skeletal muscle and meat quality are driven on the one hand by the energy reserves in the muscle and their metabolisation as well as on the other hand by the muscle structure itself.

### *cis *and *trans *mode of regulation of gene expression in QTL regions for WHC

Expression-QTL for genes showing high correlation with the phenotype may provide the necessary information required for identifying genes that control quantitative phenotypes. Categorizing eQTL has the potential to enable reverse genetics approaches for the identification of genes controlling quantitative traits, and may also help to enhance the rate of QTL cloning [50]. In particular, if the pQTL for drip loss were caused by interstrain differences of gene expression, the genetic determinants responsible for the pQTL would be restricted to the genes that were encoded inside the pQTL region and provide variations of gene expression under *cis *acting transcriptional fashion in the F2 population. In this case, their eQTL were found to reside at the same chromosomal positions at which they were encoded and the lod score curves with the peak of eQTL should coincide with those of the pQTL. Local eQTL where expression phenotypes map to the genes themselves, are of great interest, because they are direct candidates for previously mapped pQTL.

Many investigations have reported the successful mapping of quantitative trait loci for gene expression phenotypes (eQTL) in rat or mice [51-53]. Such genetical genomics analyses in livestock are still scarce. Among livestock species, poultry is well placed to embrace this technology. De Koning et al. [54] identified the *cis *and *trans *effects for a functional body weight QTL on chicken chromosome 4 in breast tissue samples from chickens with contrasting QTL genotypes. Kadarmideen and Janss [55] presented a comparative systems genetic analysis on the physiology of cortisol levels in mice and pigs with the aim to show the potential of a comprehensive computational approach to quickly identify candidate genes. Here, the first expression QTL study is presented performed in a segregating pig population with focus on the trait drip loss. In a first step we analysed the correlation between trait dependent gene expression and the phenotype drip loss, which revealed biologically meaningful relationship. In the second step, eQTL were identified for transcripts that showed trait correlated expression, which supplies us with information about the genomic location of putative regulatory loci. This strategy reduces the number of several thousand eQTLs which were not associated with drip loss. The *trans *acting eQTL represent transcripts whose abundance is regulated by loci remote from the genomic locus of each of these genes. In our study the proportion of *trans *eQTL is higher (92%) than in other studies (60%–65%) [51,56]. Here eQTL analysis was focussed on functional positional candidate genes for a trait that varies in degree, i.e. the study was driven by transcriptional and positional restrictions on the genes analysed. A network of genes relevant to the traits was addressed representing additive and pleiotropic as well as non-additive epistatic effects on the trait. This may lead to higher proportion of *trans *regulated genes compared to studies were eQTL were identified independent from any positional restrictions on the corresponding genes. *Cis *acting eQTL serve as an important new resource for the identification of positional candidates in QTL studies. We detected 8 out of 104 genes acting in *cis*, whereas Yashimita et al., [56] and Dumas et al., [57] reported 9 out of 13 genes and 1 out of 5 genes, respectively, acting in *cis*.

### Candidate genes for WHC

The candidacy of *cis *regulated functional positional candidate genes has three-fold experimental evidence. In particular for *AHNAK *a number of reasons for its candidacy for drip loss have been put forward: (i) This gene is located in the SSC2 QTL region for drip loss as confirmed by RH-mapping. The pQTL for drip in this region was also found in other studies [14,16,17]. (ii) The correlation between drip loss and *AHNAK *is high (r = 0.53; p < 0.0001). (iii) The eQTL for *AHNAK *indicates a *cis *acting mode of regulation with genome wide significance (lod score = 6.4; F = 18.2). Real time RT-PCR performed for AHNAK support the microarray data in terms of trait correlated expression. Also significant correlation was observed of expression values obtained from microarrays and real time RT-PCR, respectively. Further, eQTL analysis of real time RT-PCR data matches those of microarray data. *AHNAK *is a functional candidate gene due to its role in muscle contraction, cell adhesion and proliferation as well as its interaction with calcium. *AHNAK*, a nuclear phosphoprotein with the estimated molecular mass of 700 Da, is expressed in all muscular cells [58,59]. *AHNAK *is implicated in calcium flux regulation. At low calcium concentrations, *AHNAK *proteins are mainly localized in the nucleus, but the increase in intracellular calcium levels leads the protein to translocate to the plasma membrane [60]. *AHNAK *relocates from the cytosol to the cytosolic surface of the plasma membrane during the formation of cell-cell contacts [61]. The main localization of *AHNAK *is at the plasma membrane in adult muscle cells [59]. *AHNAK *contains three distinct structural regions: the NH_2_-terminal 251-amino acid region, a large central region of about 4300 amino acids with 36 repeated units, and the COOH-terminal 1002 amino acids region. The carboxyl-terminal region of *AHNAK *proteins mediates cellular localization and interaction with L-type Ca^2+ ^channels, calcium-binding S100B protein, as well as actin of thin filaments for muscle contraction [62-64].

*MAP4K4 *a member of the serine/threonine protein kinase family is involved in MAPK signalling for cell proliferation and differentiation as response to stressors and in cell adhesion via integrin beta 1 [65,66]. Here *MAP4K4 *appeared as a prominent candidate for drip loss. *MAP4K4 *expression is induced by TNF-alpha and promotes insulin resistance [67], whereas silencing of *MAP4K4 *prevents insulin resistance in human skeletal muscle and enhances glucose uptake [68]. This evidence promotes our finding of a positive correlation of *MAP4K4 *with drip loss. Reduced *MAP4K4 *expression, promotes glucose uptake, therefore increasing glucose content in muscle cells. By increasing energy depots in the muscle prior to slaughter, the anaerobic production of lactate *post mortem *may be delayed, thereby delaying of decline in pH and reducing drip loss.

Candidacy of *SLC3A2 *was confirmed by real time PCR. *SLC3A2 *is a member of the solute carrier family and encodes a cell surface, transmembrane protein. It associates with integrins and mediates integrin-dependent signalling related to normal cell growth. Information about function of *BBC2*, *PRCC*, *USP39*, *LOC162073 *and *COQ9 *are too limited to allow deducing functional links to the trait drip loss or other candidate genes for this trait.

## Conclusion

Analysis of trait dependent expression showed a global picture on the biological networks active *ante mortem *that affect *post mortem *processes important for final establishment of meat properties. Functional annotation of differentially expressed genes revealed the general trend of genes of cytoskeleton, cell-cell contacts and signalling including calcium signalling pathways being positively correlated whereas genes of biological networks of oxidative metabolism were negatively correlated with drip loss. Physiological studies indicated that biological processes affecting meat development are driven by the *post mortem *anoxia. Abundance and activity of enzymes and proteins of energy and calcium metabolism and proteolysis of muscle structural proteins have been shown to be major determinants with regard to the trait drip loss. The meat quality phenotype established later after slaughter depends on the transcriptome of skeletal muscle prior to slaughter and thus is already determined in living cells under genetic control. Integrating expression data with QTL analysis for the trait of interest (phene QTL, pQTL) and for gene expression levels (expression QTL, eQTL) facilitates creating a priority list of genes out of the orchestra of genes of biological networks relevant to drip for further analysis and subsequent cloning of causal genes. By combining map-based and function-driven data functional positional candidate genes could be identified. By adding data derived from eQTL analysis and matching these to the gene map and pQTL map allowed addressing genes with *trans *and *cis *mode of transcriptional control. In particular functional positional candidate genes under *cis *acting regulation are of high priority for further analysis. The first porcine eQTL-map of drip correlated transcripts in pQTL regions will facilitate cloning causal genes.

## Methods

### Animals and tissue collection

**Figure 4:**
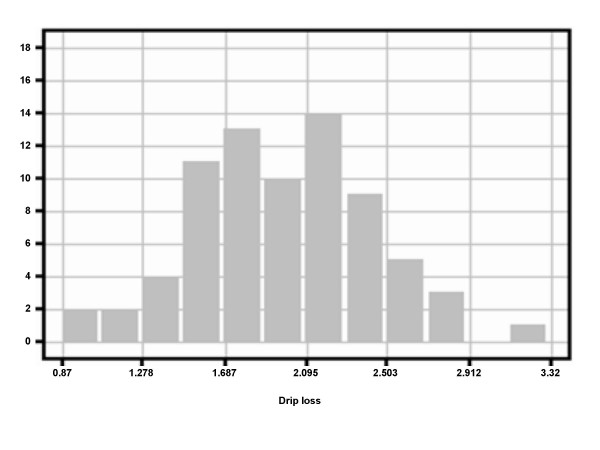
Histogram of the distribution of drip loss phenotypes among a subset of 74 animals of the DuPi population selected for chip hybridization.

This study was based on data originating from the three-generation resource family structure, trait measurements, genotyping procedures and linkage analysis as described in detail by Liu *et al*. [21]. For these experiments a total of 585 F2 progeny were used comprising 31 full-sib families. The F0 animals used were animals of two commercial breeds, the Duroc and Pietrain breed. Grandparental purebred F0 animals were reciprocal mated and 32 F1 animals were used for producing the F2. The total population was further denoted as "DuPi population". All animals were free of the mutation at the ryanodine receptor locus, RYR1, which is responsible for malignant hyperthermia syndrome. Genotypes of 116 microsatellite markers were used. For linkage mapping, twopoint and multipoint procedures of the CRI-MAP package version 2.4 were employed [69]. Expression profiling and eQTL analysis were conducted on 74 F2 animals of the resource population with previously identified pQTL for drip [21]. These 74 animals represented a subset of the population covering 25 full-sib families derived from all five F1 boars of the population and 18 out of 27 F1 sows. Animals were selected that represented the genotype combinations at the major pQTL on SSC5 and 18 at equal proportions with equal numbers of male and female [22]. Genotypes at the remaining QTL were considered as to avoid overrepresentation of any homozygote QTL genotype. As expected, when assuming mainly additive genetic effects of the QTL, the phenotype of drip loss of these selected animals had a normal distribution as shown in Figure 4.

### Drip loss phenotype

Drip loss was scored based on a bag-method using a size-standardized sample from the *longissimus dorsi *that was collected at 24 hours *post mortem*. A sample was weighed, suspended in a plastic bag, held at 4°C and re-weighed 48 hours later for water loss [70,71]. Drip loss was calculated as a percentage of lost weight based on the starting weight of a sample.

### Whole genome expression profiling

Immediately *post mortem *tissue samples were collected and snap frozen that were taken between the 13th and 14th rib from the center of *M. longissimus dorsi *of 74 animals. Total RNA was isolated using TRI Reagent (Sigma, Taufkirchen, Germany) according to the manufacturer's protocol. After DNaseI treatment the RNA was cleaned up using the RNeasy Kit (Qiagen, Hilden, Germany). The quantity of RNA was established using the NanoDrop ND-1000 spectrophotometer (Peqlab, Erlangen, Germany) and the integrity was checked by running 1 μg of RNA on 1% agarose gel. In addition absence of DNA contamination was checked using the RNA as a template in standard PCR amplifying fragments of PRL32 and HPRT. Muscle expression pattern were assessed using 74 Porcine Genome Array which contains 23,937 probe sets that interrogate approximately 23,256 transcripts from 20,201 S. scrofa genes. Preparation of target products, hybridization and scanning using the GeneChip scanner 3000 were performed according to Affymetrix protocols using 5 μg of total RNA to prepare antisense biotinylated RNA. The quality of hybridization was assessed in all samples following the manufacturer's recommendations. Data were analysed with Affymetrix GCOS 1.1.1 software using global scaling to a target signal of 500. Data were then imported into Arrays Assist software (Stratagene Europe, Amsterdam, The Netherlands) for subsequent analysis. The data were processed with MAS5.0 to generate cell intensity files (present or absent). Quantitative expression levels of the present transcripts were estimated using PLIER (Probe Logarithmic Intensity Error) for normalization. The microarray data related to all samples have been deposited in the Gene Expression Omnibus (GEO, [72]) public repository (GEO accession number: GSE10204).

### Correlation between drip loss phenotype and gene expression

Phenotypic data, i.e. expression levels and drip loss, were adjusted for systematic effects by analysis of variance performed with the procedure 'Mixed' of the SAS software package (SAS System for Windows, Release 8.02) before analysing their correlation and performing eQTL analysis. Full-sib family and sex were used as fixed effects, carcass weight as a covariate and slaughter date as random effects. Pearson correlation coefficients were calculated between the predicted values of the log2 transformed expression intensities of all 11,453 probes and the predicted values of drip loss of the 74 animals used. For each pair of transcript and drip loss, Pearson correlation together with the P-value was computed. The corresponding q-values were calculated to determine the FDR [73]. Genes that showed correlation at r ≥ 0.37 with p ≤ 0.001, corresponding to q ≤ 0.004, were analyzed further.

### Functional annotation clustering

Based on BLAST comparison of EnsEMBL human cDNA and genomic sequences with the Affymetrix porcine target sequences, which were extended with porcine sequence information of the Pig Gene Index (Institute for Genome Research, TIGR), 19,675 of 24,123 transcripts on the Affymetrix Porcine microarray, representing 11,265 unique genes, were annotated [23]. This source of annotation list was used in this study. In addition, probe sets showing trait dependent expression with bit scores below 50 were rechecked for their identity by blasting Affymetrix core sequences of these probe sets before functional annotation analysis. The list of significantly trait correlated transcripts was analyzed according to predefined pathways and functional categories annotated by KEGG [28], and GO [74] using the DAVID bioinformatic resource [75]. Therefore, the Affymetrix IDs of the human probe sets corresponding to the porcine probes sets were used as reported by Tsai et al., [23]. By this, differentially regulated genes were functionally annotated to large amounts of physiological pathway information that are of general relevance. However, physiology of some cellular processes may differ between species. Pig physiology closely resembles human physiology, thus given the lack of porcine-specific pathways the use the human pathways information and extrapolation of these pathways for the pig can be expected to provide biological meaningful results [76].

### eQTL analysis

In order to map eQTL adjusted expression values of 1279 unique probe sets showing significant correlation to drip loss were regressed onto the additive and dominance coefficients in intervals of 1 cM using the F2 option of QTL express [77]. Chromosome-wide and genome-wide significance levels were estimated by permutation tests [29]. The analysis identified 897 eQTL with chromosome-wide and genome-wide significance. Mapping of eQTL to the gene itself indicates that *cis *changes are responsible for the different expression levels, whereas mapping positions of eQTL different from the position of the corresponding genes indicate *trans *regulation. Correspondingly, *cis *acting regulation of transcription was considered for genes where available published experimental mapping data or comparative mapping data indicated their position within the corresponding interval of flanking markers of the eQTL peak; genes mapping outside the flanking marker interval of their corresponding eQTL were considered having *trans *acting regulation of transcription.

### Mapping of *AHNAK*

Mapping of *AHNAK *was achieved by screening of the Radiation Hybrid mapping panel of INRA, France, and Minnesota University, USA, IMpRH, and analysis of the data vector using the two-point and the multi-point analysis option of the IMpRH mapping tool [78].

### Quantitative Real-time PCR (qRT-PCR)

Transcripts of *AHNAK *and *SLC3A2 *were quantified by real-time reverse transcription PCR (RT-PCR) using the iCycler apparatus (Bio-Rad Laboratories GmbH, Munich, Germany) and the iQ SYBR Green Supermix (Bio-Rad). Real time RT-PCR were performed in duplicate using 56 animals of 22 full-sib families out of 74 individuals of 25 full-sib families that were previously used for microarray analysis. RNA was isolated as described above. Two microgram RNA were reverse transcribed to cDNA using SuperScriptIII MMLV reverse transcriptase (Invitrogen, Karlsruhe, Germany) in a reaction containing 500 ng oligo (dT)_11_VN primer 500 ng random hexamer primer according to the manufacturer's protocol. Templates were amplified using the gene specific primers (AHNAKup 3'-tgtcactggctcaccagaag-5', AHNAKdw 3'-gtcgctgaaggaatttgagc-5' and SLC3A2up 3'-ctgtggctgccaagatgaag-5', SLC3A2dw 3'-atctgctgtaggtcggagga-5') by 45 cycles of 95°C for 15 seconds denaturation, 60°C for 30 seconds annealing, and 72°C for 30 seconds extension preceded by initial denaturation of 95°C for 10 minutes as a universal thermal cycling parameter. Based on the analysis of melting curves of the PCR products a high temperature fluorescence acquisition point was estimated and included to the amplification cycle program. For all assays a standard curve was generated by amplifying serial dilutions of specific PCR products. After completion of the qPCR melting curve analysis and afterwards agarose gel electrophoresis were performed to confirm specificity of the amplification. Normalisation of variation in RT-PCR efficiency and initial RNA input was performed by using *RPL32 *(RPL32up 3'-agcccaagatcgtcaaaaag-5'; RPL32dw 3'-tgttgctcccataaccaatg-5') and *HPRT***(**HPRTup 3'-acactggcaaaacaatgcaa-5'; HPRTdw 3'-tcaagggcatagcctaccac-5') gene as internal standard and by dividing calculated *AHNAK *and *SLC3A2 *mRNA copy numbers with a mean normalization factor derived from the expression of the reference genes. Real time RT-PCR and microarray data were compared by Pearson correlation (SAS version 9.1 SAS Institute, Cary, NC) and eQTL were estimated as described above.

## Abbreviations

QTL: quantitative trait loci; eQTL: expression quantitative trait loci; pQTL: phene quantitative trait loci; SSC: Sus scrofa chromosome; PLIER: Probe Logarithmic Error Intensity Estimate; GO: Gene Ontology; KEGG: Kyoto Encyclopedia of Genes and Genomes; FDR: false discovery ratio;

## Authors' contributions

SP analyzed the microarray data and wrote the paper; EJ and CP collected the material and analyzed the linkage map; EM, MS, TS and CW aided in data analysis and helped in drafting the manuscript; KS and KW conceived and designed the study, contributed to data interpretation and helped in drafting the manuscript.

## Supplementary Material

Additional File 1 Coefficients of correlation (r) between drip loss and expression level, p values and q value. the table lists the Affymetrix probe set IDs with coefficients of correlation (r) of expression level and the trait drip loss as well as corresponding p-values and q-values. Click here for file

Additional File 2 897 eQTL with chromosome-wide significance at p ≤ 0.05 including 68 eQTL significant at p ≤ 0.01 chromosome-wide level and 48 and 12 eQTL significant at genome-wide p ≤ 0.05 and p ≤ 0.01 significance levels, coefficients of correlation (r) between drip loss and expression level, p values and q values. the table lists the subset of Affymetrix probe set IDs with information about eQTL detected at different levels of significance. Click here for file

Additional File 3 104 significant eQTL, which were detected in QTL regions for drip loss on SSC 2, 3, 4, 5,6 and 18; coefficients of correlation (r) between drip loss and expression level, p values and q values. the table lists the subset of Affymetrix probe set IDs with eQTL detected within QTL regions for the trait drip loss. Click here for file

Additional File 4 66 candidate genes mapping within the pQTL regions for drip loss on SSC 2, 3, 4, 5, 6 and 18 but showing 119 eQTL in other genomic region; coefficients of correlation (r) between drip loss and expression level, p values and q values. the table lists the subset of Affymetrix probe set IDs that map within QTL regions for the trait drip loss but show eQTL outside any QTL region for drip loss. Click here for file
